# Bacteriome Diversity of Soil Islands Associated With Bromeliads From Ironstone Outcrops in the Brazilian Pantanal

**DOI:** 10.1155/ijm/6374781

**Published:** 2025-07-28

**Authors:** Fernanda M. R. Godoy, Gecele M. Paggi, Aline P. Lorenz, Jeferson V. Ramos, Daniel G. Franco, Fernando M. L. Calarge, Nayara F. L. Garcia, Marcus V. S. Urquiza, Jolimar A. Schiavo, Nalvo F. Almeida, Marivaine S. Brasil

**Affiliations:** ^1^Microbiology and Genetics Laboratory, Pantanal Campus, Federal University of Mato Grosso do Sul, Corumbá, Mato Grosso do Sul, Brazil; ^2^Ecology and Evolutionary Biology Laboratory, Institute of Biosciences, Federal University of Mato Grosso do Sul, Campo Grande, Mato Grosso do Sul, Brazil; ^3^Ecology Laboratory, Pantanal Campus, Federal University of Mato Grosso do Sul, Corumbá, Mato Grosso do Sul, Brazil; ^4^Department of Soils, State University of Mato Grosso do Sul, Aquidauana, Mato Grosso do Sul, Brazil; ^5^Faculty of Computing, Federal University of Mato Grosso do Sul, Campo Grande, Mato Grosso do Sul, Brazil

**Keywords:** 16S rRNA gene, Bromeliaceae, eDNA, metabarcoding

## Abstract

Knowledge about the diversity and distribution of microorganisms in natural environments is essential for understanding the dominant microbial groups and predicting their ecological functions. This study is aimed at describing the bacteriome diversity in soils associated with bromeliads in the Brazilian Pantanal region, utilizing genomic approaches. We analyzed the 16S rRNA gene from soil environmental DNA (eDNA) samples linked to *Bromelia balansae* and *Deuterocohnia meziana* (Bromeliaceae), which inhabit ironstone outcrops in the Pantanal. The analysis revealed Ktedonobacteraceae as the most abundant bacterial group, showing a mean relative abundance of 22.8% ± 15.5% in *B. balansae* and 33.5% ± 18.4% in *D. meziana* soils. Other highly abundant families were Chthoniobacteraceae and Pyrinomonadaceae, each exceeding 14.5% mean abundance. Despite the similarities in bacteriome composition between the bromeliads, beta-diversity analysis revealed phylogenetic distinctions across localities. The São João and Vale do Paraíso Farms, which experience the highest human impact from livestock farming, showed considerable differences, with 25 and 13 exclusive taxa, respectively. The environmental stresses of ironstone outcrops, such as high insolation and thermal variation, likely favor specific taxa adapted to these conditions. Understanding the bacteriome diversity in these unique habitats is crucial for promoting sustainable use and conserving the Pantanal's biodiversity.

## 1. Introduction

Microbial communities have multiple biological functions and play a key role in biogeochemical cycles. They are involved in the cycling of organic compounds, influence above-ground ecosystems, and contribute to plant nutrition, health, and structure, and soil fertility [1–3], being an important indicator of soil quality and providing information for its assessment [3]. In the past few decades, the development of genomic approaches has shown great potential for identifying the diversity of microbial communities [4, 5], enabling scientists to describe ecosystem diversity and facilitate comparisons between microbiomes, providing valuable scientific results [5]. Many studies have shown the effectiveness of molecular techniques such as those utilizing environmental DNA (eDNA) for assessing the structure and diversity of microbial communities [5–7] by sequencing of 16S rRNA gene hypervariable regions to identify the bacteriome [5, 7–9].

Recent studies have shown that soil microbial diversity is influenced by several factors, such as acidity, texture, the amount of organic matter, the availability of nutrients (nitrogen and phosphorus (P)), and climatic factors such as temperature, humidity, and seasonality [10, 11]. In addition, soil microbial diversity varies significantly among different biomes, such as tropical and temperate forests, savannas, prairies, tundra, deserts, and aquatic ecosystems, depending on local environmental factors [10, 12–14]. These studies also suggest that the plant–microbiome relationship varies among different taxonomic groups and spatial scales [15, 16], highlighting the need for a comprehensive analysis that considers data from taxonomic groups and biomes to elucidate the relationship between plant and microbial diversity.

The soil microbial community forms a complex system supporting and regulating plant growth, playing essential roles in mineral solubilization and the fixation of vital nutrients such as nitrogen and iron, as well as in the degradation of organic compounds like cellulose [17, 18]. This microbiota also produces metabolites, such as auxins, which promote root development and contribute to increased plant biomass [18]. Additionally, soil microorganisms perform critical functions in carbon dioxide capture, enhancing soil quality [18]. This potential allows for the use of microorganisms in phytoremediation, promoting the decomposition and degradation of environmental toxins [18], as well as the oxidation of potentially contaminant compounds, such as iron [19] and manganese [20].

**Figure 1:**
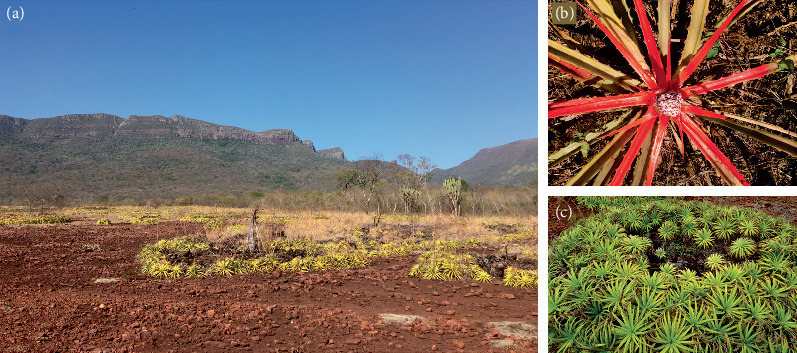
(a) Ironstone outcrop in the Vale do Paraíso Farm showing soil islands with *Deuterocohnia meziana* in the Urucum Residual Plateau, Corumbá city, MS, Brazil. (b) Detail of a flowering *Bromelia balansae* individual. (c) Detail of a group of *Deuterocohnia meziana* individuals.

In Brazil, one of the least studied biomes is the ironstone outcrops, also known as “cangas” or petroplinthite, which occur mainly in the Central Plateau and the Northeast Region of Brazil. In the Amazon region, it is more widely distributed as plinthite (nonhardened laterite) [21]. The Quadrilátero Ferrífero (Minas Gerais state [MG]), Serra de Carajás (Pará state [PA]), Caetité (Bahia state [BA]), and Urucum Residual Plateau (URP) (Mato Grosso do Sul state [MS]) retain the largest reserves of iron ore in Brazil [22]. Unfortunately, these regions are subjected to economic exploitation activities without proper environmental planning, which has been the main threat to these habitats [23]. The URP (MS) is a region formed by ironstone outcrops, also known as lateritic benches [24] (Figure 1a) or “canga couraçada” [25], consisting of hardened lateritic material that is rich in iron.

The URP, together with the limestone hills of Corumbá city, is one of the residual hill formations located along the Bolivian border, west of the Paraguay River, with a maximum altitude of 1060 m, constituting the highest point of the Pantanal region, MS, Brazil, covering an area of 1311 km^2^ (Figure 1a), and covering a high richness flora [26]. The ironstone outcrops of the URP are in drainage areas (approximately 100 m altitude) at the foot of the hills; they present a small slope [25], with little aptitude for agricultural or pastoral uses [26]. These hardened ferruginous substrates can be considered ecosystems similar to those in outcrops where vascular plants establish themselves directly on the hardened substrate or between rock fragments (saxicolous plants) or in the form of clusters referred to in several studies as soil islands [27, 28]. Despite its ecological importance, these ecosystems are among the least studied and most threatened in Brazil due to their association with high-quality iron ore deposits and economic exploitation [28].

Studies on the microbial communities associated with plant species that occur in ore deposit regions are limited, and in the face of the continuous pressure of extractive action, some species, together with their microbiota, are being decimated [4]. The bacterial community plays important ecological roles, such as facilitating plant growth or conferring protection against desiccation, maintaining the survival of plants on ferruginous and rocky substrates [4]. Knowledge about the diversity and distribution of microorganisms in natural environments is critical for understanding the evolutionary processes responsible for the selection of dominant groups. In addition, identifying the main bacterial groups in different areas may help predict the ecological functions that these microorganisms mediate. Thus, the main objective of this study was to describe the bacteriome diversity of soil islands associated with the bromeliad species *Bromelia balansae* Mez and *Deuterocohnia meziana* Kuntze ex Mez (Bromeliaceae) using eDNA soil samples from ironstone outcrops in the URP, MS, Brazil. Based on the bacteriome diversity results, we discussed the characteristics related to the functional and ecological roles of the bacterial groups identified in this environment.

## 2. Material and Methods

### 2.1. Soil Sampling and eDNA Extraction

**Figure 2:**
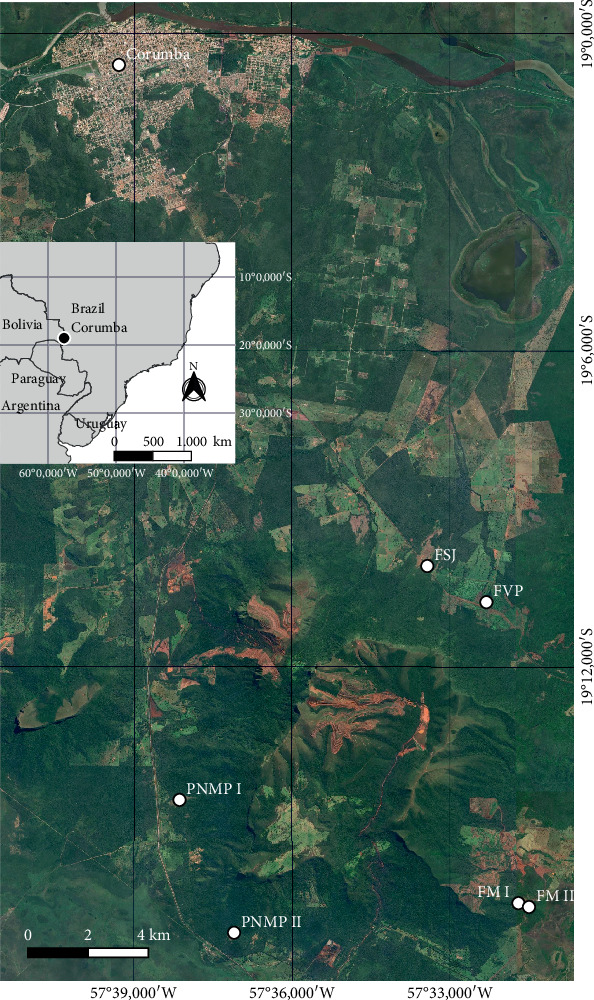
Map of the study area in the Corumbá city, Mato Grosso do Sul State, Brazil. Location of the collecting areas in the Pantanal region is shown with white bullets followed by the locality ID: Monjolinho Farm I (FM I), Monjolinho Farm II (FM II), Vale do Paraíso Farm (FVP), Piraputangas Municipality Natural Park I (PMNP I), Piraputangas Municipality Natural Park II (PMNP II), and São João Farm (FSJ).

This study was conducted on ironstone outcrops at the URP, Corumbá, MS, Brazil (Figure 1a). The climate is classified as Aw by the Köppen system, with dry winters and rainy summers [29]. The mean annual precipitation is 1070 mm in the dry season, which lasts from May to September; the average monthly temperature is 25°C, ranging from 21.4°C to 27.7°C [29]. During sampling in August 2021, the local temperature varied between 24°C and 26°C. The study species *B. balansae* Mez and *D. meziana* Kuntze *ex* Mez (Bromeliaceae) occur on ironstone outcrops at the URP (MS), forming groups on soil islands (Figure 1b,c) [30]. eDNA soil samples were collected from six different ironstone outcrops around the URP: Monjolinho Farm I (FM I), Monjolinho Farm II (FM II), São João Farm (FSJ), Vale do Paraíso Farm (FVP), Piraputangas Municipality Natural Park I (PMNP I), and Piraputangas Municipality Natural Park II (PMNP II) (Figure 2 and Table 1).

The soil above the roots of the bromeliads at a depth of 0–5 cm was collected in triplicate using an auger at each sampling location. The triplicate samples were mixed in the laboratory to create one composite sample. During sampling at FVP, it was observed that the area was recently burned. Soil samples were sent for physicochemical analysis at the Soil Laboratory of the State University of Mato Grosso do Sul (UEMS). Afterward, the samples were weighed, and eDNA was extracted using a DNeasy PowerSoil (QIAGEN) kit according to the manufacturer's instructions.

### 2.2. 16S rRNA Gene Metabarcoding Sequencing

16S rRNA gene amplification targeting the V4 region was performed in triplicates using the primers 515F (5⁣′ - GTGCCAGCMGCCGCGGTAA -3⁣′) and 806R (3⁣′ - GGACTACHVGGGTWTCTAAT -5⁣′) [31, 32]. The PCR mixture consisted of 22.5 *μ*L of Platinum PCR SuperMix High Fidelity (Thermo Fisher), 2 *μ*L of genomic DNA (~20 ng), and 0.5 *μ*L of each primer (10 *μ*M). The PCR amplification consisted of an initial temperature of 94°C for 3 min, followed by 40 cycles of denaturation at 94°C for 30 s, primers annealing at 60°C for 30 s, and extension at 68°C for 1 min. PCR triplicates were united and purified with AMPure XP reagent (Beckman Coulter, Indianapolis, IN, United States) in two rounds. The samples were quantified in a Qubit instrument and then diluted to 40 pM. The amplicon pool was loaded onto the Ion Chef System (Thermo Fisher Scientific) for emulsion PCR, enrichment, and loading onto an Ion S5 530 chip. Following templating and chip loading, the samples were sequenced using 850 nucleotide (*π*) flows in the Ion GeneStudio S5 System, following the manufacturer's instructions (Life Technologies). The sequence data were deposited at NCBI SRA under the BioProject accession number PRJNA1063691, and specific BioSample accession numbers are presented in Table 1.

### 2.3. Sequence and Statistical Analysis

Bacteriome analyses were performed using QIIME2 2023.9.1 [33]. The raw sequence data were quality filtered by denoising with DADA2 [34]. All amplicon sequence variants (ASVs) were aligned with MAFFT [35] and used to construct a phylogenetic tree with FastTree 2 [36]. The ASV table was normalized to relative abundance. For alpha-diversity metrics, we used Shannon's and Faith's diversities [37], and for beta-diversity metrics, we used principal coordinate analysis (PCoA) to visualize sample dissimilarities considering the Jaccard distance for qualitative measures and Bray–Curtis dissimilarity for quantitative measures [38]. We also used weighted UniFrac [39] for quantitative measure of community dissimilarity and unweighted UniFrac [40] for qualitative measures (phylogenetic relationships). The Kruskal–Wallis test was used to statistically compare alpha-diversity metrics from areas and species samples. PERMANOVA of beta-diversity metrics using 999 permutations was used to statistically compare distances between group samples.

Taxonomy was assigned to ASVs using the Bayes taxonomy classifier [41] against the Silva SSU 138 database [42]. The fractions of shared and exclusive variants were visualized using Venn diagrams (InteractiVenn web platform; http://www.interactivenn.net/) [43]. The *π* and haplotype (*h*) diversities [44] were estimated using DNASP 6 [45] and Arlequin 3.5 [46]. The Wilcoxon nonparametric test was employed to evaluate the families exhibiting differential abundance between bromeliads. Finally, the metabolic functions of the bacteria present in each sample were inferred using the FAPROTAX [47] pipeline.

## 3. Results

### 3.1. Soil Analysis, Bacteriome Composition, and Alpha-Diversity Analysis

The analysis of the soil physicochemical properties revealed acidic to neutral pH values ranging from 4 to 7 (Table 2). FM I and PMNP II localities had the greatest amounts of nutrients (P, K, Ca, and Mg) when compared to the other locations sampled. FVP had the highest P content, probably due to a recent burn (Table 2).

We obtained a total of 1137.416 sequences from 11 eDNA soil samples, six from *B. balansae* and five from *D. meziana*. We generated 14,166 ASVs from these sequences, hereafter called features. Soil samples from *B. balansae* produced a total of 8296 ASVs and 6758 from *D. meziana*. Altogether, these ASVs represented a total of 42 identified phyla and one unidentified bacterial phylum (Table S1). Ktedonobacteraceae (Chloroflexi) was the most abundant bacterial family for both bromeliads, with a mean relative abundance of 22.8% ± 15.5% for *B. balansae* and 33.5% ± 18.4% for *D. meziana*. Chthoniobacteraceae (Verrucomicrobiota) and Pyrinomonadaceae (Acidobacteria) were also abundant families, with relative abundances greater than 14.5%.

**Figure 3:**
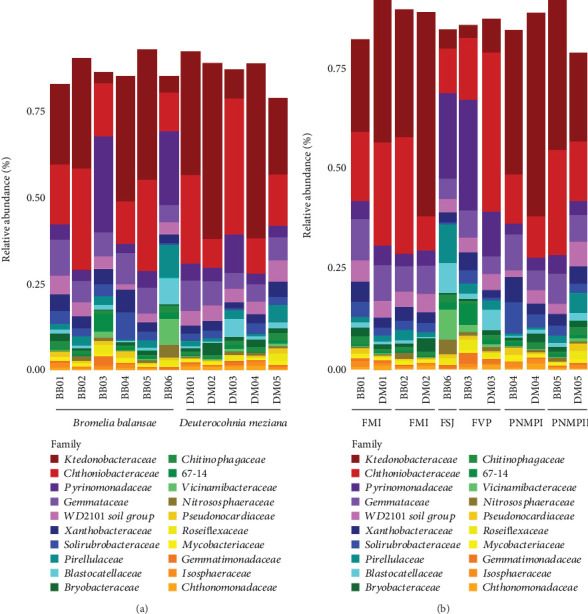
Bacteriome diversity composition at the 20 most abundant families identified by DADA2 in RStudio. The data are presented for (a) the species, *Bromelia balansae* and *Deuterocohnia meziana*. The stacked bars are representations of each sample, and colored fragments represent the fraction of each sample assigned to each bacterial family. (b) The localities Monjolinho Farm I (FM I), Monjolinho Farm II (FM II), São João Farm (FSJ), Vale do Paraíso Farm (FVP), Piraputangas Municipality Natural Park I (PMNP I), and Piraputangas Municipality Natural Park II (PMNP II).

**Figure 4:**
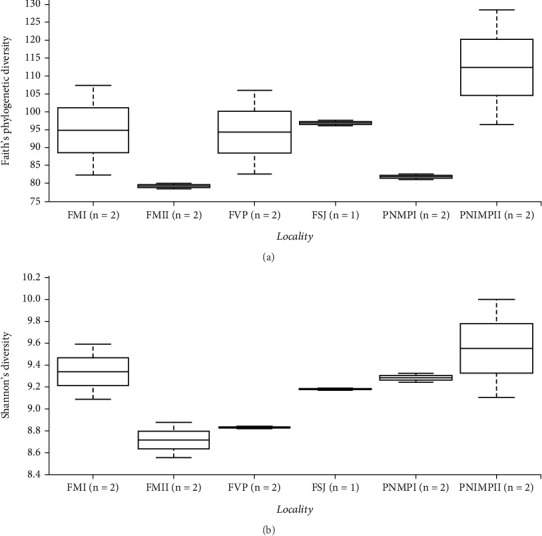
Alpha-diversity significance plots by locality. (a) Phylogenetic richness (Faith's phylogenetic diversity) (*H* = 7.63; *p* = 0.177) and (b) Shannon's diversity (*H* = 7.23; *p* = 0.204) were not significantly different between species or “Cangas” localities (*p* > 0.05).

Although no visual differences in bacterial relative abundance were observed between bromeliads, a significant shift in community dominance was evident across the various “cangas” localities (Figures 3 and 4). At FM I and II and PMNP I and II, the family Ktedonobacteraceae exhibited a greater relative abundance (mean ± SD = 32.15% ± 6.28%). In contrast, at FVP and FSJ, the dominant families were Chthoniobacteraceae (29.4%) and Pyrinomonadaceae (21.4%), with Ktedonobacteraceae showing a reduced relative abundance (6 ± 1%). By genus, the most predominant was *Udaeobacter* (24.3% ± 9.74%; Verrucomicrobiota); *RB41* (13.4% ± 12.3%; Acidobacteria); and *HSB OF53-F07* (9.97% ± 5.54%; Chloroflexi).

We observed a slight difference in the maximum number of features between the bromeliads but a greater difference in the “cangas” localities (Figure 4a,b). A greater number of features as a metric of richness were found in PMNP II and FM I, followed by FVP, FSJ, and PMNP I and FM II. However, the phylogenetic richness and Shannon's diversity did not significantly differ between species or “cangas” localities (*p* > 0.05; Figure 4; Tables S2 and S3).

### 3.2. Beta-Diversity Analysis and Taxa Exclusivity

**Figure 5:**
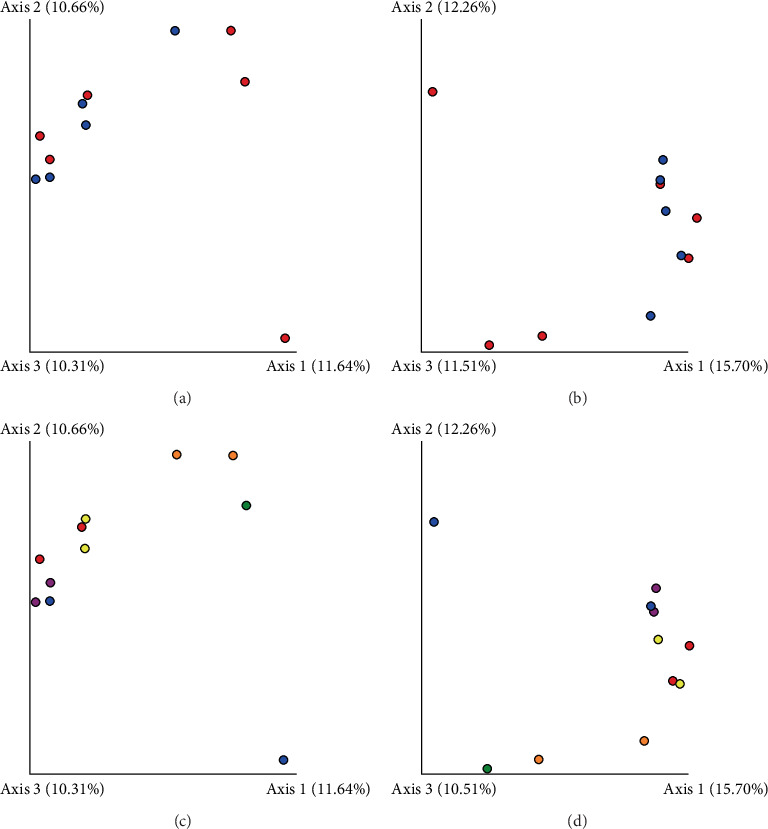
Similarities among bacterial communities from *Bromelia balansae* (red) and *Deuterocohnia meziana* (blue) from six different ironstone outcrops, FM I (red), FM II (blue), FSJ (green), FVP (orange), PMNP I (purple), and PMNP II (yellow). (a) Jaccard by species. (b) Bray–Curtis by species. (c) Jaccard by localities. (d) Bray–Curtis by localities. The similarities were calculated using the Jaccard abundance in the QIIME2 program.

**Figure 6:**
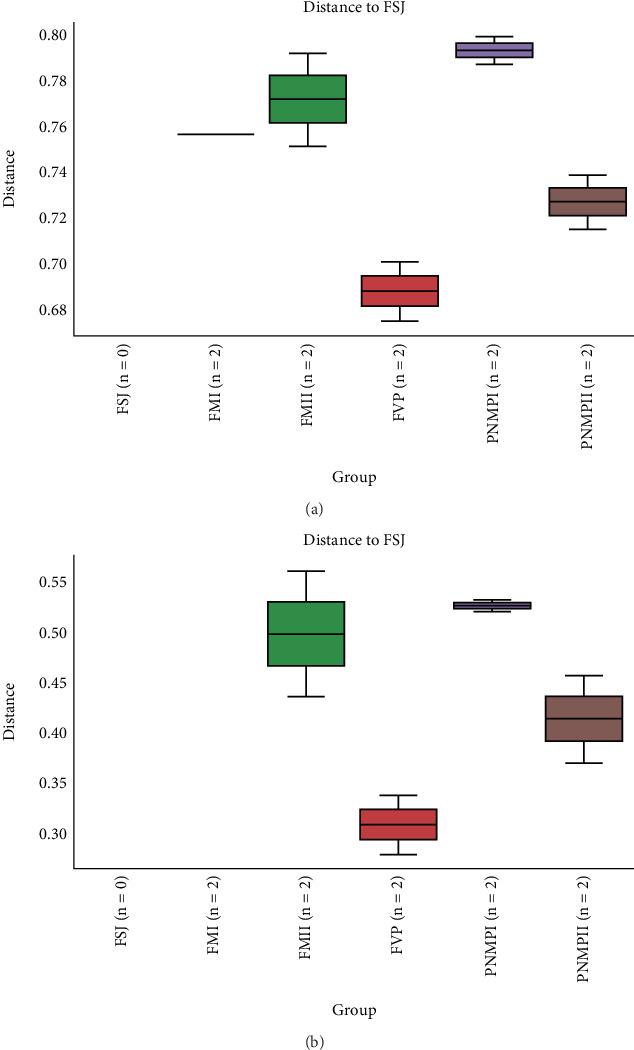
Beta-diversity metrics (from QIIME2). Beta-diversity group significance using (a) unweighted UniFrac distance from the FSJ to the other localities, based on the ASV count (test statistic = 1.341; *p* = 0.005) and (b) weighted UniFrac distance from the FVP to other localities, based on the ASV count (test statistic = 2.099; *p* = 0.042).

Based on comparisons of the bromeliad species bacteriomes, the results from the PERMANOVA test revealed no significant differences in any of the beta-diversity metrics (Figures 5a, 5b, 5c, and 5d). The PCoA results (Jaccard distance) revealed that FSJ and FVP localities were the most different (Figure 5c). Besides, “cangas” locality significantly explained the unweighted UniFrac distance (Figure 6a; PERMANOVA, *p* < 0.05), suggesting that the distances are more related to phylogenetic relationships since the weighted UniFrac distance analysis was also significant (Figure 6b; PERMANOVA, *p* < 0.05). Exclusive low-abundance taxa play a significant role in UniFrac distance, and this metric structure may explain the significantly different bacteriome compositions among “cangas” localities. Despite the significant differences between unweighted and weighted UniFrac distances, there was no statistically significant difference according to pairwise comparisons (Tables S4 and S5).

**Figure 7:**
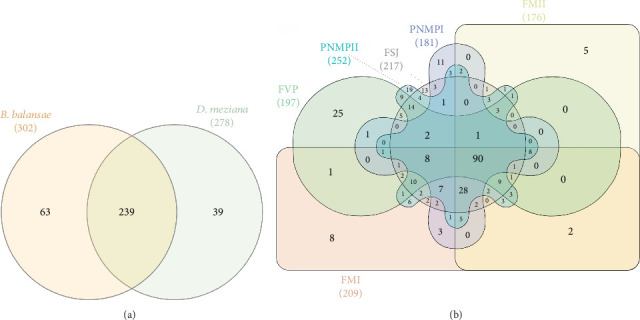
Venn diagram for each subgroup, (a) species and (b) locality. Together with the name of the subgroup, the total number of variants represented is indicated in parentheses. The number of variants that are shared between the subgroups is shown at the intersections of the sets.

A total of 239 bacterial groups were shared between the *B. balansae* and *D. meziana* soils, with a total of 63 and 39 exclusive taxa, respectively (Figure 7a; Tables S6 and S7). The six areas analyzed shared a total of 90 identified taxa (Figure 7b), FM I and FM II localities had eight and five exclusive taxa, respectively, and FVP had a total of 25 exclusive taxa (Table S8). Of the 217 taxa identified in FSJ, only 13 were exclusive, and PMNP I and PMNP II had 11 and 17 exclusive taxa, respectively (Figure 7b; Table S8).

### 3.3. Genetics and Functional Diversity and Relative Abundance of Bacteria Families

**Figure 8:**
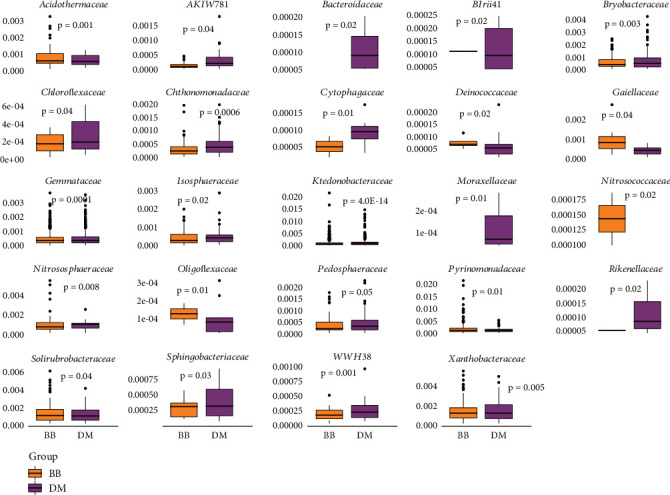
Boxplot showing the relative abundance of the 24 microbial families that showed statistical significance in the Wilcoxon test for the soil microbiota associated with *Bromelia balansae* and *Deuterocohnia meziana*. Differences in abundance between the two species were assessed using the Wilcoxon test, with significant *p* values indicated on the graph.

Acidobacteria, Chloroflexi, had the highest values for *h* and *π* diversities (Table 3), and Verrucomicrobiota, with the lowest number of ASVs, also presented the lowest values of genetic diversities. There was a differential abundance of bacteria associated with *B. balansae* and *D. meziana*, regarding 24 families (Figure 8). Eight families showed higher abundance in *B. balansae* and 13 for *D. meziana*. The families *Bacteroidaceae* and *Moraxellaceae* were exclusive to *D. meziana*, whereas *Nitrosococcaceae* was identified exclusively in *B. balansae* (Table S9).

**Figure 9:**
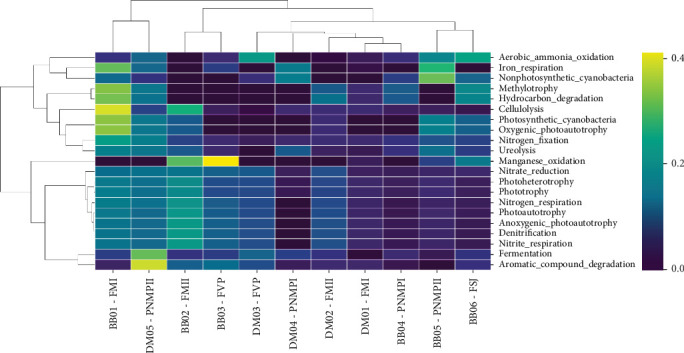
Heatmap of bacterial metabolic functions. Data were obtained based on the occurrence of ASVs (number of ASVs capable of each function) derived from samples of *Bromelia balansae* and *Deuterocohnia meziana*.

The heat map of functional diversity (Figure 9) revealed a significant increase in functions related to denitrification in samples of *B. balansae* at the FM I, FM II, and FVP sites and in *D. meziana* at the FM II, PMNP II, and FVP sites. This increase can be attributed to the rise in abundance of *Rubrobacter* and *Rhodoplanes*, important for nitrate reduction. An increase in iron respiration was noted for *D. meziana* at the PMNP II and PMNP I sites, as well as in *B. balansae* at the FM I, FSJ, and PMNP II sites, due to the presence of *Acidicaldus*. A significant increase in the abundance of the genus *Geodermatophilus*, known for manganese oxidation, was also observed in *B. balansae* at the PMNP II, FSJ, FM II, and especially at the FVP sites. Notably, an increase of *Acidothermus* was recorded at the FM I and FM II sites for *B. balansae*, a cellulolytic taxon.

## 4. Discussion

Soil microorganisms play essential roles in maintaining the balance and health of the soil ecosystem [17, 18]. Bacteria, for instance, contribute to plant growth regulation, enhance the absorption and processing of nutrients in the soil, and synthesize phytohormones that support plant development [18]. This study presents important results concerning the bacteriome diversity of ironstone outcrops (“cangas”) and the dominant bacterial groups in the soil associated with bromeliad species. The URP is threatened by mining and livestock activities, which modify the physicochemical properties of the soil, and consequently, the bacteriome composition from each locality, considering the specific groups (qualitative) and their relative abundances (quantitative).

The alpha-diversity in our study showed no differences, indicating the similar richness and uniformity of soil bacteria on ironstone outcrops. This result may be related to soil bacterial colonization strategies, where some bacteria have large populations with greater dispersal capacity, or related to resource restrictions, such as available nutrients [48]. Considering the beta-diversity results, the FSJ and FVP localities were the most distinct among all the sampled locations. Currently, due to the expansion of livestock farming, both locations have been used to establish pastures through clearing and burning vegetation (personal observation) which may explain the high levels of P observed in FVP (Table 2) and the differences from the other undisturbed localities [49–51]. Environmental and anthropogenic factors can lead to the loss of diversity, as they ultimately shape the microbial community by causing physical changes in the soil, which influence the availability of nutrients and ions, in addition to influencing biogeochemical processes, such as the inhibition of microbiological nitrification [49]. Among the environmental and anthropogenic factors, the removal of vegetation cover, establishment of pastures, entry of pollutants, and agricultural management are important [49–51]. In addition to the threats that cause environmental degradation and affect diversity levels, the lack of knowledge about bacteriome diversity itself is also a factor contributing to diversity loss since we cannot protect what is unknown. Identifying the main bacterial groups in different areas may provide an opportunity to discover novel taxa with specific ecological functions potentially applicable to biotechnological processes, agriculture, healthcare, and industry.

The presence of *Udaeobacter*, the most abundant genera, has been previously reported in the literature [52]; it has the ability to absorb nutrients released during the lysis of other microorganisms, thereby reducing the need for the energetically costly synthesis of biomolecules, which may explain its higher prevalence compared to other genera [52]. Additionally, *Udaeobacter* plays a role in the oxidation of H_2_, a fundamental step in the hydrogen cycle [52]. Hydrogen present in the soil acts as a regulatory factor in pH control and contributes to environmental homeostasis [18]. Furthermore, *Udaeobacter* is known to produce secondary metabolites with antibiotic properties, which can induce significant changes in the bacterial composition of the soil [52]. The genus *RB41*, the second most abundant, plays a crucial role in maintaining metabolism and biogeochemical functions, especially under prolonged low-nutrient stress conditions [53, 54]. This taxon shows resilience to adverse soil conditions and is essential for nutrient cycling, contributing to the decomposition of organic matter and the mineralization of carbon and nitrogen, being fundamental to preserving biodiversity and maintaining soil ecosystem stability [53, 54]. Therefore, the abundance of this genus in the “canga” soils is notable, which are characterized by low nutrient availability and organic matter content, requiring organisms adapted to sustain nutrient cycling and decomposition processes in such harsh habitats. The microbial interaction leads to the release of nutrients such as P, potassium, and iron, along with the production of organic acids that aid in their solubilization [17]. The activity of denitrifying bacteria is particularly crucial for nitrogen cycling and availability in the soil, as evidenced by the abundance of genera *Rubrobacter* and *Rhodoplanes* [18].

A significant abundance of the genus *Geodermatophilus* was observed in *B. balansae* samples. *Geodermatophilus* is predominantly found in desert environments, adapted to extreme conditions of high UV radiation and low humidity [20], exactly the conditions observed in the “cangas.” These bacteria have the capacity to oxidize manganese, a process that leads to the formation of rock varnish, characteristic of arid regions, providing protective and stabilizing functions for the soil. Additionally, *Geodermatophilus* species perform important ecological roles, contributing to soil formation and nutrient cycling, including carbon and nitrogen assimilation [20]. Our findings indicate an increase in the abundance of *Acidicaldus*, commonly found in extreme environments, such as mining areas and acidic sediments, where it stands out for its ability to oxidize sulfur and iron compounds, contributing to nutrient cycling and heavy metal detoxification, playing a crucial role in the biogeochemistry of these ecosystems, and acting as an important agent in the bioremediation of contaminated areas [19, 55]. The pH is a crucial factor that influences the structure of bacterial communities in soil. In “cangas” soils, it is already known that pH tends to be low, around 4.0 [56]. In the present study, the pH of the sampled areas ranged from 4 to 7 (Table 2). Goffredi et al. [57] investigated tank-forming bromeliads and observed that pH is determinate in the bacterial composition. In bromeliad tanks with pH below 5.1, the phyla Alphaproteobacteria, Acidobacteria, and Planctomycetes predominated, while in environments with pH above 5.3, communities were dominated by Betaproteobacteria and Firmicutes. Our data revealed that Chloroflexi, Verrucomicrobia, and Acidobacteria were the most abundant phyla, followed by Planctomycetes, Actinobacteria, and Proteobacteria (Figure 3), according to previous initial results from our research group [58].

This study revealed the taxonomic composition of microbial communities present in soils from ironstone outcrops associated with two bromeliad species. The data demonstrated that certain microbial groups are favored by environmental conditions such as thermal amplitude, acidic pH, and nutrient availability. Our data revealed that the soil bacteriome can be different between disturbed and undisturbed areas. This study provides important information about the structure of the bacteriome community from ironstone outcrop areas. Finally, the results reported here expand the current knowledge of bacteriome taxonomy in ironstone outcrops, contributing to understanding the diversity of soil bacteriomes around the world, aiming for their sustainable use and conservation.

## Figures and Tables

**Table 1:** Locality, ID, geographical coordinates, bromeliad species, sample code, and GenBank BioSample accession numbers of soil samples collected from ironstone outcrops associated with *Bromelia balansae* and *Deuterocohnia meziana* in the Urucum Residual Plateau, Corumbá city, Mato Grosso do Sul, Brazil.

**Locality**	**Geographical coordinates**	**Species**	**Sample code**	**BioSample accession numbers**
Monjolinho Farm I (FM I)	19°16⁣′28.4⁣^″^ S, 57°31⁣′42.7⁣^″^ W	*Bromelia balansae*	BB01	SAMN39403979
		*Deuterocohnia meziana*	DM01	SAMN39403980

Monjolinho Farm II (FM II)	19°16⁣′32.80⁣^″^ S, 57°31⁣′30.69⁣^″^ W	*Bromelia balansae*	BB02	SAMN39403981
		*Deuterocohnia meziana*	DM02	SAMN39403982

Vale do Paraíso Farm (FVP)	19°10⁣′46.2⁣^″^S, 57°32⁣′19.1⁣^″^ W	*Bromelia balansae*	BB03	SAMN39403983
		*Deuterocohnia meziana*	DM03	SAMN39403984

Piraputangas Municipality Natural Park I (PMNP I)	19°14⁣′31.0⁣^″^ S, 57°38⁣′08.0⁣^″^ W	*Bromelia balansae*	BB04	SAMN39403985
		*Deuterocohnia meziana*	DM04	SAMN39403986

Piraputangas Municipality Natural Park II (PMNP II)	19°17⁣′02.0⁣^″^ S, 57°37⁣′06.0⁣^″^ W	*Bromelia balansae*	BB05	SAMN39403987
		*Deuterocohnia meziana*	DM05	SAMN39403988

São João Farm (FSJ)	19°10⁣′05.1⁣^″^ S, 57°33⁣′26.3⁣^″^ W	*Bromelia balansae*	BB06	SAMN39403989

**Table 2:** Physicochemical properties of soil samples. Localities identification (ID), *Bromelia balansae* (*BB*), and *Deuterocohnia meziana* (*DM*).

**ID species**	**FM I (*BB*)**	**FM I (*DM*)**	**FM II (*BB*)**	**FM II (*DM*)**	**FVP (*BB*)**	**FVP (*DM*)**	**FSJ (*BB*)**	**PMNP I (*BB*)**	**PMNP I (*DM*)**	**PMNP II (*BB*)**	**PMNP II (*DM*)**
pH	4.8	4.2	5.1	4.4	4.3	4.7	4.1	6.2	5.5	7	4
P	22.4	22.3	62.9	9.5	28	194.9	20.7	9	57.4	11.2	48.3
K	0.27	0.3	0.36	0.13	0.32	0.21	0.09	0.25	0.29	0.31	0.21
Na	0.054	0.069	0.084	0.056	0.122	0.053	0.066	0.139	0.002	0.104	0.053
Ca	7.64	2.31	9.58	3.9	4.02	4.56	1.49	9.02	9.78	13.09	2.46
Mg	1.83	0.55	1.44	1.04	0.73	0.31	0.34	3.47	1.13	3.86	0.43
H + Al	11.7	18.5	10.2	10.6	14.8	19.6	15.8	1.2	6	1	19.7
Al	0.41	1.34	0.21	0.52	0.52	1.13	1.24	0.05	0.1	0	1.44
SB	9.7	3.2	11.4	5.1	5.1	5.1	1.9	12.7	11.2	17.3	3.1
*V* (%)	45.3	14.6	52.7	32.5	25.5	20.6	10.8	91.5	65.3	94.4	13.6
*m* (%)	1.92	6.17	0.96	3.3	2.6	4.6	6.97	0.37	0.6	0	6.32

*Note:* Ca, Na, Mg, K, Al, potential acidity (H + Al), and sum of bases (SB) are expressed in cmol_c_/dm^3^. P is expressed in mg dm^3^. P, Na, and K are Mehlich 1 extractors.

Abbreviations: H + Al, potential acidity; *m*, Al saturation index (%); *V*, base saturation index (%).

**Table 3:** Haplotype (*h*) and nucleotide (*π*) diversity of the ASVs from the most abundant phyla in the bacterioma considering all soil samples, which were collected at ironstone outcrops associated with *Bromelia balansae* and *Deuterocohnia meziana* in the Urucum Residual Plateau, Corumbá city, Mato Grosso do Sul, Brazil.

**Phyla**	**No. reads**	**No. of ASVs**	**No. polymorphic sites**	**h**	**π**
Acidobacteria	169,242	1189	218	0.9959 (± 0.0000)	0.150226 (± 0.071998)
Chloroflexi	298,552	3484	374	0.9983 (± 0.0000)	0.157962 (± 0.075365)
Verrucomicrobiota	171,288	616	209	0.9557 (± 0.00003)	0.065837 (± 0.032224)

## Data Availability

The 16S rRNA gene data are available at the NCBI Sequence Read Archive (SRA) under the bioproject PRJNA1063691. Metadata will be made available from the corresponding author upon reasonable request.

## Supporting Information

Supporting Information Additional supporting information can be found online in the Supporting Information section. Table S1: Top 20 most abundant bacterial taxa. The values represent the relative abundance (%) of each genus in each sample. Table S2: Statistical comparison of localities considering phylogenetic richness (Faith's phylogenetic diversity) and Kruskal–Wallis (pairwise) test, implemented by QIIME2. Table S3: Statistical comparison of localities considering Shannon's diversity and Kruskal–Wallis (pairwise) test, implemented by QIIME2. Table S4: Overview and pairwise PERMANOVA results from unweighted-UniFrac analysis for localities. Table S5: Overview and pairwise PERMANOVA results from weighted-UniFrac analysis for localities. Table S6: Exclusive bacteriome taxa from soil associated with *Bromelia balansae* from ironstone outcrops. Samples were collected in Corumbá city, MS, Brazil. k, kingdom; p, phylum; c, class; o,order; f, family. Table S7: Exclusive bacteriome taxa from soil associated with *Deuterochonia meziana* from ironstone outcrops. Samples were collected in Corumbá city, MS, Brazil. k, kingdom; p, phylum; c, class; o, order; f, family. Table S8: Exclusive bacteriome taxa for localities considering both species, *Bromelia balansae* and *Deuterochonia meziana*. Localities: Monjolinho Farm I (FM I), Monjolinho Farm II (FM II), São João Farm (FSJ), Vale do Paraíso Farm (FVP), Piraputangas Municipality Natural Park I (PMNP I), and Piraputangas Municipality Natural Park II (PMNP II). Samples were collected in Corumbá city, MS, Brazil. k, kingdom; p, phylum; c, class; o, order; f, family. Table S9: Differential abundance analysis was performed using the Wilcoxon test. The values presented correspond to the *p* values calculated for each sample and location.
