# Supplementary material for: Bacteriome Diversity of Soil Islands Associated With Bromeliads From Ironstone Outcrops in the Brazilian Pantanal
Source: Int J Microbiol. 2025 Jul 28;2025:6374781. doi: 10.1155/ijm/6374781 (PMC12321424; doi:10.1155/ijm/6374781)
Supplement: Supporting Information — Additional supporting information can be found online in the Supporting Information section. Table S1: Top 20 most abundant bacterial taxa. The values represent the relative abundance (%) of each genus in each sample. Table S2: Statistical comparison of localities considering phylogenetic richness (Faith's phylogenetic diversity) and Kruskal–Wallis (pairwise) test, implemented by QIIME2. Table S3: Statistical comparison of localities considering Shannon's diversity and Kruskal–Wallis (pairwise) test, implemented by QIIME2. Table S4: Overview and pairwise PERMANOVA results from unweighted-UniFrac analysis for localities. Table S5: Overview and pairwise PERMANOVA results from weighted-UniFrac analysis for localities. Table S6: Exclusive bacteriome taxa from soil associated with Bromelia balansae from ironstone outcrops. Samples were collected in Corumbá city, MS, Brazil. k, kingdom; p, phylum; c, class; o,order; f, family. Table S7: Exclusive bacteriome taxa from soil associated with Deuterochonia meziana from ironstone outcrops. Samples were collected in Corumbá city, MS, Brazil. k, kingdom; p, phylum; c, class; o, order; f, family. Table S8: Exclusive bacteriome taxa for localities considering both species, Bromelia balansae and Deuterochonia meziana. Localities: Monjolinho Farm I (FM I), Monjolinho Farm II (FM II), São João Farm (FSJ), Vale do Paraíso Farm (FVP), Piraputangas Municipality Natural Park I (PMNP I), and Piraputangas Municipality Natural Park II (PMNP II). Samples were collected in Corumbá city, MS, Brazil. k, kingdom; p, phylum; c, class; o, order; f, family. Table S9: Differential abundance analysis was performed using the Wilcoxon test. The values presented correspond to the p values calculated for each sample and location. [file 6374781.f1.docx]

**Bacteriome diversity of soil islands associated with bromeliads from ironstone outcrops in the Brazilian Pantanal**

**Fernanda M. R. Godoy^1^ | Gecele M. Paggi^2^ | Aline P. Lorenz^2^ | Jeferson V. Ramos^2^ | Daniel G. Franco^2^ | Fernando M. L. Calarge^2^ | Nayara F. L. Garcia^1^ | Marcus V. S. Urquiza^3^ | Jolimar A. Schiavo^4^ | Nalvo F. Almeida^5^ | Marivaine S. Brasil^1^**

*^1^Microbiology and Genetics Laboratory, Pantanal Campus, Federal University of Mato Grosso do Sul, Corumbá, Mato Grosso do Sul, Brazil*

*^2^Ecology and Evolutionary Biology Laboratory, Institute of Biosciences, Federal University of Mato Grosso do Sul, Campo Grande, Mato Grosso do Sul, Brazil*

*^3^Ecology Laboratory, Pantanal Campus, Federal University of Mato Grosso do Sul, Corumbá, Mato Grosso do Sul, Brazil*

*^4^Department of Soils, State University of Mato Grosso do Sul, Aquidauana, Mato Grosso do Sul, Brazil*

*^5^Faculty of Computing, Federal University of Mato Grosso do Sul, Campo Grande, Mato Grosso do Sul, Brazil*

**Correspondence**

Gecele Matos Paggi, Ecology and Evolutionary Biology Laboratory, Institute of Biosciences, Federal University of Mato Grosso do Sul, Av. Costa e Silva, s/nº, Campo Grande, 79070-900, MS, Brazil

Email: [gecele.paggi@ufms.br](mailto:gecele.paggi@ufms.br)

**Supplementary Information**

Supplementary Table 1: Top 20 most abundant bacterial taxa. The values represent the relative abundance (%) of each genus in each sample.

<https://docs.google.com/spreadsheets/d/1OGGiE_a32HWIced4FhBaxVHaRdaAo3Ya/edit?usp=sharing&ouid=101432216138639808492&rtpof=true&sd=true>

Supplementary Table 2: Statistical comparison of localities considering phylogenetic richness (Faith’s Phylogenetic Diversity), Kruskal-Wallis (pairwise) test, implemented by Qiime2.

|  |  | **H** | **p-value** | **q-value** |
| --- | --- | --- | --- | --- |
| **Group 1** | **Group 2** |  |  |  |
| **FM I (n=2)** | **FM II (n=2)** | 2.4 | 0.121335 | 0.260004 |
|  | **FVP (n=2)** | 0.0 | 1.000000 | 1.000000 |
|  | **FSJ (n=1)** | 0.0 | 1.000000 | 1.000000 |
|  | **PNMP I (n=2)** | 2.4 | 0.121335 | 0.260004 |
|  | **PNMP II (n=2)** | 0.6 | 0.438578 | 0.598061 |
| **FM II (n=2)** | **FVP (n=2)** | 2.4 | 0.121335 | 0.260004 |
|  | **FSJ (n=1)** | 1.5 | 0.220671 | 0.367786 |
|  | **PNMP I (n=2)** | 2.4 | 0.121335 | 0.260004 |
|  | **PNMP II (n=2)** | 2.4 | 0.121335 | 0.260004 |
| **FVP (n=2)** | **FSJ (n=1)** | 0.0 | 1.000000 | 1.000000 |
|  | **PNMP I (n=2)** | 2.4 | 0.121335 | 0.260004 |
|  | **PNMP II (n=2)** | 0.6 | 0.438578 | 0.598061 |
| **FSJ (n=1)** | **PNMP I (n=2)** | 1.5 | 0.220671 | 0.367786 |
|  | **PNMP II (n=2)** | 0.0 | 1.000000 | 1.000000 |
| **PNMP I (n=2)** | **PNMP II (n=2)** | 2.4 | 0.121335 | 0.260004 |

Supplementary Table 3: Statistical comparison of localities considering Shannon’s Diversity, Kruskal-Wallis (pairwise) test, implemented by Qiime2.

|  |  | **H** | **p-value** | **q-value** |
| --- | --- | --- | --- | --- |
| **Group 1** | **Group 2** |  |  |  |
| **FM I (n=2)** | **FM II (n=2)** | 2.4 | 0.121335 | 0.303338 |
|  | **FVP (n=2)** | 2.4 | 0.121335 | 0.303338 |
|  | **FSJ (n=1)** | 0.0 | 1.000000 | 1.000000 |
|  | **PNMP I (n=2)** | 0.0 | 1.000000 | 1.000000 |
|  | **PNMP II (n=2)** | 0.6 | 0.438578 | 0.657867 |
| **FM II (n=2)** | **FVP (n=2)** | 0.0 | 1.000000 | 1.000000 |
|  | **FSJ (n=1)** | 1.5 | 0.220671 | 0.367786 |
|  | **PNMP I (n=2)** | 2.4 | 0.121335 | 0.303338 |
|  | **PNMP II (n=2)** | 2.4 | 0.121335 | 0.303338 |
| **FVP (n=2)** | **FSJ (n=1)** | 1.5 | 0.220671 | 0.367786 |
|  | **PNMP I (n=2)** | 2.4 | 0.121335 | 0.303338 |
|  | **PNMP II (n=2)** | 2.4 | 0.121335 | 0.303338 |
| **FSJ (n=1)** | **PNMP I (n=2)** | 1.5 | 0.220671 | 0.367786 |
|  | **PNMP II (n=2)** | 0.0 | 1.000000 | 1.000000 |
| **PNMP I (n=2)** | **PNMP II (n=2)** | 0.0 | 1.000000 | 1.000000 |

Supplementary Table 4: Overview and pairwise PERMANOVA results from unweighted-unifrac analysis for localities.

**Overview**

|  | **PERMANOVA results** |
| --- | --- |
| **method name** | PERMANOVA |
| **test statistic name** | pseudo-F |
| **sample size** | 11 |
| **number of groups** | 6 |
| **test statistic** | 1.341045 |
| **p-value** | 0.005 |
| **number of permutations** | 999 |

**Pairwise permanova results**

| **Group 1** | **Group 2** | **Sample size** | **Permutations** | **pseudo-F** | **p-value** | **q-value** |
| --- | --- | --- | --- | --- | --- | --- |
| **FM I** | **FM II** | 4 | 999 | 0.899963 | 1.000 | 1.000000 |
|  | **FVP** | 4 | 999 | 1.341647 | 0.363 | 0.458750 |
|  | **FSJ** | 3 | 999 | 1.789942 | 0.342 | 0.458750 |
|  | **PNMP I** | 4 | 999 | 1.114145 | 0.327 | 0.458750 |
|  | **PNMP II** | 4 | 999 | 0.880900 | 0.674 | 0.722143 |
| **FM II** | **FVP** | 4 | 999 | 1.517187 | 0.367 | 0.458750 |
|  | **FSJ** | 3 | 999 | 1.752490 | 0.317 | 0.458750 |
|  | **PNMP I** | 4 | 999 | 1.131713 | 0.320 | 0.458750 |
|  | **PNMP II** | 4 | 999 | 1.028861 | 0.662 | 0.722143 |
| **FVP** | **FSJ** | 3 | 999 | 1.353707 | 0.348 | 0.458750 |
|  | **PNMP I** | 4 | 999 | 1.801958 | 0.318 | 0.458750 |
|  | **PNMP II** | 4 | 999 | 1.150194 | 0.361 | 0.458750 |
| **FSJ** | **PNMP I** | 3 | 999 | 2.287230 | 0.321 | 0.458750 |
|  | **PNMP II** | 3 | 999 | 1.362443 | 0.333 | 0.458750 |
| **PNMP I** | **PNMP II** | 4 | 999 | 1.309348 | 0.354 | 0.458750 |

Supplementary Table 5: Overview and pairwise PERMANOVA results from weighted-unifrac analysis for localities.

**Overview**

|  | **PERMANOVA results** |
| --- | --- |
| **method name** | PERMANOVA |
| **test statistic name** | pseudo-F |
| **sample size** | 11 |
| **number of groups** | 6 |
| **test statistic** | 2.099735 |
| **p-value** | 0.042 |
| **number of permutations** | 999 |

**Pairwise permanova results**

| **Group 1** | **Group 2** | **Sample size** | **Permutations** | **pseudo-F** | **p-value** | **q-value** |
| --- | --- | --- | --- | --- | --- | --- |
| **FM I** | **FM II** | 4 | 999 | 0.670262 | 1.000 | 1.000 |
|  | **FSJ** | 4 | 999 | 3.004839 | 0.330 | 0.549 |
|  | **FVP** | 3 | 999 | 5.100252 | 0.339 | 0.549 |
|  | **PNMP I** | 4 | 999 | 1.268987 | 0.348 | 0.549 |
|  | **PNMP II** | 4 | 999 | 0.503488 | 0.664 | 0.830 |
| **FM II** | **FSJ** | 4 | 999 | 2.969250 | 0.331 | 0.549 |
|  | **FVP** | 3 | 999 | 3.099392 | 0.366 | 0.549 |
|  | **PNMP I** | 4 | 999 | 0.689674 | 1.000 | 1.000 |
|  | **PNMP II** | 4 | 999 | 0.759384 | 1.000 | 1.000 |
| **FVP** | **FSJ** | 3 | 999 | 1.149478 | 0.641 | 0.830 |
|  | **PNMP I** | 4 | 999 | 4.180060 | 0.341 | 0.549 |
|  | **PNMP II** | 4 | 999 | 1.707197 | 0.332 | 0.549 |
| **FSJ** | **PNMP I** | 3 | 999 | 5.432382 | 0.348 | 0.549 |
|  | **PNMP II** | 3 | 999 | 2.239025 | 0.319 | 0.549 |
| **PNMP I** | **PNMP II** | 4 | 999 | 1.403497 | 0.325 | 0.549 |

Supplementary Table 6: Exclusive bacteriome taxa from soil associated with *Bromelia balansae* from ironstone outcrops. Samples were collected in Corumbá city, MS, Brazil. k = kingdom; p = phylum; c = class; o = order; f = family.

| **Exclusive bacteriome taxa** |
| --- |
| k__Bacteria;p__Acidobacteria;c__[Chloracidobacteria];__;__ |
| k__Bacteria;p__Acidobacteria;c__Acidobacteria-6;__;__ |
| k__Bacteria;p__Acidobacteria;c__Acidobacteria-6;o__iii1-15;f__RB40 |
| k__Bacteria;p__Acidobacteria;c__BPC102;o__MVS-40;f__ |
| k__Bacteria;p__Acidobacteria;c__Holophagae;o__Holophagales;f__Holophagaceae |
| k__Bacteria;p__Acidobacteria;c__RB25;o__;f__ |
| k__Bacteria;p__Acidobacteria;c__S035;o__;f__ |
| k__Bacteria;p__Actinobacteria;c__Acidimicrobiia;o__Acidimicrobiales;f__AKIW874 |
| k__Bacteria;p__Actinobacteria;c__Actinobacteria;o__Actinomycetales;f__Tsukamurellaceae |
| k__Bacteria;p__Actinobacteria;c__MB-A2-108;o__0319-7L14;f__ |
| k__Bacteria;p__Actinobacteria;c__Thermoleophilia;o__Gaiellales;f__AK1AB1_02E |
| k__Bacteria;p__Armatimonadetes;c__SJA-176;o__GAB-B06;f__ |
| k__Bacteria;p__Bacteroidetes;c__[Saprospirae];o__[Saprospirales];f__ |
| k__Bacteria;p__Bacteroidetes;c__[Saprospirae];o__[Saprospirales];f__Saprospiraceae |
| k__Bacteria;p__Bacteroidetes;c__Bacteroidia;o__Bacteroidales;f__ |
| k__Bacteria;p__Chlamydiae;c__Chlamydiia;o__Chlamydiales;f__Simkaniaceae |
| k__Bacteria;p__Chlorobi;c__SJA-28;o__;f__ |
| k__Bacteria;p__Chloroflexi;c__Anaerolineae;o__Ardenscatenales;f__Ardenscatenaceae |
| k__Bacteria;p__Chloroflexi;c__Anaerolineae;o__GCA004;f__ |
| k__Bacteria;p__Chloroflexi;c__Anaerolineae;o__H39;f__ |
| k__Bacteria;p__Chloroflexi;c__Anaerolineae;o__S0208;f__ |
| k__Bacteria;p__Chloroflexi;c__Anaerolineae;o__SHA-20;f__ |
| k__Bacteria;p__Chloroflexi;c__Chloroflexi;o__Chloroflexales;__ |
| k__Bacteria;p__Chloroflexi;c__Gitt-GS-136;o__;f__ |
| k__Bacteria;p__Chloroflexi;c__SAR202;o__;f__ |
| k__Bacteria;p__Chloroflexi;c__TK10;o__AKYG885;f__ |
| k__Bacteria;p__Cyanobacteria;c__;o__;f__ |
| k__Bacteria;p__FCPU426;c__;o__;f__ |
| k__Bacteria;p__Firmicutes;c__Bacilli;o__Lactobacillales;f__Streptococcaceae |
| k__Bacteria;p__Firmicutes;c__Clostridia;o__Clostridiales;f__Symbiobacteriaceae |
| k__Bacteria;p__Firmicutes;c__Clostridia;o__Clostridiales;f__Veillonellaceae |
| k__Bacteria;p__Gemmatimonadetes;c__Gemm-2;o__;f__ |
| k__Bacteria;p__Gemmatimonadetes;c__Gemm-3;o__;f__ |
| k__Bacteria;p__GOUTA4;c__;o__;f__ |
| k__Bacteria;p__OD1;c__ZB2;o__;f__ |
| k__Bacteria;p__Planctomycetes;__;__;__ |
| k__Bacteria;p__Planctomycetes;c__;o__;f__ |
| k__Bacteria;p__Planctomycetes;c__C6;o__MVS-107;f__ |
| k__Bacteria;p__Planctomycetes;c__OM190;o__CL500-15;f__ |
| k__Bacteria;p__Planctomycetes;c__Phycisphaerae;__;__ |
| k__Bacteria;p__Planctomycetes;c__Phycisphaerae;o__CCM11a;f__ |
| k__Bacteria;p__Planctomycetes;c__Pla3;o__;f__ |
| k__Bacteria;p__Planctomycetes;c__vadinHA49;o__DH61;f__ |
| k__Bacteria;p__Proteobacteria;c__Alphaproteobacteria;o__RF32;f__ |
| k__Bacteria;p__Proteobacteria;c__Alphaproteobacteria;o__Rhizobiales;f__Phyllobacteriaceae |
| k__Bacteria;p__Proteobacteria;c__Alphaproteobacteria;o__Rhodospirillales;f__ |
| k__Bacteria;p__Proteobacteria;c__Alphaproteobacteria;o__Rickettsiales;f__Rickettsiaceae |
| k__Bacteria;p__Proteobacteria;c__Betaproteobacteria;o__A21b;f__EB1003 |
| k__Bacteria;p__Proteobacteria;c__Betaproteobacteria;o__Neisseriales;f__Neisseriaceae |
| k__Bacteria;p__Proteobacteria;c__Deltaproteobacteria;o__Desulfobacterales;f__Desulfobulbaceae |
| k__Bacteria;p__Proteobacteria;c__Deltaproteobacteria;o__NB1-j;f__NB1-i |
| k__Bacteria;p__Proteobacteria;c__Gammaproteobacteria;o__Alteromonadales;f__Alteromonadaceae |
| k__Bacteria;p__Proteobacteria;c__Gammaproteobacteria;o__Legionellales;f__Coxiellaceae |
| k__Bacteria;p__Spirochaetes;c__[Leptospirae];o__[Leptospirales];f__Sediment-4 |
| k__Bacteria;p__Spirochaetes;c__Spirochaetes;o__Spirochaetales;f__Spirochaetaceae |
| k__Bacteria;p__Synergistetes;c__Synergistia;o__Synergistales;f__Dethiosulfovibrionaceae |
| k__Bacteria;p__Tenericutes;c__Mollicutes;o__Acholeplasmatales;f__Acholeplasmataceae |
| k__Bacteria;p__TM7;c__TM7-3;o__EW055;f__ |
| k__Bacteria;p__Verrucomicrobia;c__[Pedosphaerae];o__[Pedosphaerales];f__OPB35 |
| k__Bacteria;p__WS2;c__SHA-109;o__;f__ |
| k__Bacteria;p__WS3;c__PRR-12;o__Sediment-1;f__ |
| k__Bacteria;p__WS3;c__PRR-12;o__Sediment-1;f__CV106 |
| k__Bacteria;p__WS3;c__PRR-12;o__wb1_H11;f__ |

Supplementary Table 7: Exclusive bacteriome taxa from soil associated with *Deuterochonia meziana* from ironstone outcrops. Samples were collected in Corumbá city, MS, Brazil. k = kingdom; p = phylum; c = class; o = order; f = family.

| **Exclusive bacteriome taxa** |
| --- |
| k__Bacteria;p__Actinobacteria;c__Actinobacteria;o__Actinomycetales;f__Cellulomonadaceae |
| k__Bacteria;p__Actinobacteria;c__Actinobacteria;o__Actinomycetales;f__Corynebacteriaceae |
| k__Bacteria;p__Actinobacteria;c__Actinobacteria;o__Actinomycetales;f__Intrasporangiaceae |
| k__Bacteria;p__Bacteroidetes;c__At12OctB3;o__;f__ |
| k__Bacteria;p__Bacteroidetes;c__Bacteroidia;o__Bacteroidales;f__Bacteroidaceae |
| k__Bacteria;p__Bacteroidetes;c__Bacteroidia;o__Bacteroidales;f__Rikenellaceae |
| k__Bacteria;p__Bacteroidetes;c__Cytophagia;o__Cytophagales;f__[Amoebophilaceae] |
| k__Bacteria;p__Chloroflexi;c__Chloroflexi;o__Chloroflexales;f__ |
| k__Bacteria;p__Chloroflexi;c__Chloroflexi;o__Herpetosiphonales;f__ |
| k__Bacteria;p__Chloroflexi;c__Ktedonobacteria;o__Thermogemmatisporales;f__ |
| k__Bacteria;p__Chloroflexi;c__SHA-26;o__;f__ |
| k__Bacteria;p__Chloroflexi;c__TK10;o__AKYG885;__ |
| k__Bacteria;p__Cyanobacteria;c__Nostocophycideae;o__Nostocales;f__Scytonemataceae |
| k__Bacteria;p__Cyanobacteria;c__Oscillatoriophycideae;o__Oscillatoriales;f__Phormidiaceae |
| k__Bacteria;p__Firmicutes;__;__;__ |
| k__Bacteria;p__Firmicutes;c__Bacilli;o__Bacillales;f__Thermoactinomycetaceae |
| k__Bacteria;p__Firmicutes;c__Bacilli;o__Lactobacillales;f__Lactobacillaceae |
| k__Bacteria;p__Firmicutes;c__Clostridia;o__Clostridiales;__ |
| k__Bacteria;p__Firmicutes;c__Clostridia;o__Clostridiales;f__Lachnospiraceae |
| k__Bacteria;p__Firmicutes;c__Clostridia;o__Clostridiales;f__Peptococcaceae |
| k__Bacteria;p__Gemmatimonadetes;c__Gemmatimonadetes;__;__ |
| k__Bacteria;p__Gemmatimonadetes;c__Gemmatimonadetes;o__Gemmatimonadales;f__Ellin5301 |
| k__Bacteria;p__Planctomycetes;c__Phycisphaerae;o__Pla1;f__ |
| k__Bacteria;p__Proteobacteria;c__Alphaproteobacteria;o__BD7-3;f__ |
| k__Bacteria;p__Proteobacteria;c__Alphaproteobacteria;o__Rhizobiales;f__Xanthobacteraceae |
| k__Bacteria;p__Proteobacteria;c__Alphaproteobacteria;o__Rickettsiales;__ |
| k__Bacteria;p__Proteobacteria;c__Betaproteobacteria;__;__ |
| k__Bacteria;p__Proteobacteria;c__Betaproteobacteria;o__SC-I-84;f__ |
| k__Bacteria;p__Proteobacteria;c__Deltaproteobacteria;__;__ |
| k__Bacteria;p__Proteobacteria;c__Deltaproteobacteria;o__Myxococcales;f__0319-6G20 |
| k__Bacteria;p__Proteobacteria;c__Gammaproteobacteria;o__;f__ |
| k__Bacteria;p__Proteobacteria;c__Gammaproteobacteria;o__Pseudomonadales;f__Moraxellaceae |
| k__Bacteria;p__Proteobacteria;c__TA18;o__CV90;f__ |
| k__Bacteria;p__TM7;c__;o__;f__ |
| k__Bacteria;p__TM7;c__TM7-3;__;__ |
| k__Bacteria;p__Verrucomicrobia;__;__;__ |
| k__Bacteria;p__Verrucomicrobia;c__[Methylacidiphilae];o__Methylacidiphilales;f__ |
| k__Bacteria;p__Verrucomicrobia;c__Opitutae;o__HA64;f__ |
| k__Bacteria;p__WS5;c__;o__;f__ |

Supplementary Table 8: Exclusive bacteriome taxa for localities considering both species, *Bromelia balansae* and *Deuterochonia meziana*. Localities: Monjolinho Farm I (FM I), Monjolinho Farm II (FM II), São João Farm (FSJ), Vale do Paraíso Farm (FVP), Piraputangas Municipality Natural Park I (PNMP I), and Piraputangas Municipality Natural Park II (PNMP II). Samples were collected in Corumbá city, MS, Brazil. k = kingdom; p = phylum; c = class; o = order; f = family.

| **Locality** | **Exclusive bacteriome taxa** |
| --- | --- |
| **FM I** | k__Bacteria;p__Chloroflexi;c__SAR202;o__;f__ |
|  | k__Bacteria;p__Planctomycetes;c__vadinHA49;o__DH61;f__ |
|  | k__Bacteria;p__Proteobacteria;c__Alphaproteobacteria;o__RF32;f__ |
|  | k__Bacteria;p__Proteobacteria;c__Alphaproteobacteria;o__Rhizobiales;f__Phyllobacteriaceae |
|  | k__Bacteria;p__Proteobacteria;c__Alphaproteobacteria;o__Rickettsiales;f__Rickettsiaceae |
|  | k__Bacteria;p__Proteobacteria;c__Betaproteobacteria;__;__ |
|  | k__Bacteria;p__Proteobacteria;c__Betaproteobacteria;o__A21b;f__EB1003 |
|  | k__Bacteria;p__Proteobacteria;c__Gammaproteobacteria;o__;f__ |
| **FM II** | k__Bacteria;p__Actinobacteria;c__Actinobacteria;o__Actinomycetales;f__Cellulomonadaceae |
|  | k__Bacteria;p__Bacteroidetes;c__[Saprospirae];o__[Saprospirales];f__ |
|  | k__Bacteria;p__Firmicutes;__;__;__ |
|  | k__Bacteria;p__OD1;c__ZB2;o__;f__ |
|  | k__Bacteria;p__TM7;c__TM7-3;o__EW055;f__ |
|  | k__Bacteria;p__Acidobacteria;c__[Chloracidobacteria];__;__ |
|  | k__Bacteria;p__Armatimonadetes;c__SJA-176;o__GAB-B06;f__ |
|  | k__Bacteria;p__Chloroflexi;c__Anaerolineae;o__SHA-20;f__ |
|  | k__Bacteria;p__Chloroflexi;c__Chloroflexi;o__Chloroflexales;f__ |
|  | k__Bacteria;p__Chloroflexi;c__Chloroflexi;o__Herpetosiphonales;f__ |
|  | k__Bacteria;p__Chloroflexi;c__TK10;o__AKYG885;__ |
| **FSJ** | k__Bacteria;p__Chloroflexi;c__TK10;o__AKYG885;f__ |
|  | k__Bacteria;p__Firmicutes;c__Clostridia;o__Clostridiales;f__Symbiobacteriaceae |
|  | k__Bacteria;p__Gemmatimonadetes;c__Gemmatimonadetes;__;__ |
|  | k__Bacteria;p__Planctomycetes;c__Phycisphaerae;__;__ |
|  | k__Bacteria;p__TM7;c__TM7-3;__;__ |
|  | k__Bacteria;p__Verrucomicrobia;c__[Methylacidiphilae];o__Methylacidiphilales;f__ |
|  | k__Bacteria;p__WS5;c__;o__;f__ |
| **FVP** | k__Bacteria;p__Acidobacteria;c__Acidobacteria-6;__;__ |
|  | k__Bacteria;p__Acidobacteria;c__Acidobacteria-6;o__iii1-15;f__RB40 |
|  | k__Bacteria;p__Acidobacteria;c__BPC102;o__MVS-40;f__ |
|  | k__Bacteria;p__Acidobacteria;c__RB25;o__;f__ |
|  | k__Bacteria;p__Actinobacteria;c__Acidimicrobiia;o__Acidimicrobiales;f__AKIW874 |
|  | k__Bacteria;p__Bacteroidetes;c__[Saprospirae];o__[Saprospirales];f__Saprospiraceae |
|  | k__Bacteria;p__Chlorobi;c__SJA-28;o__;f__ |
|  | k__Bacteria;p__Chloroflexi;c__Anaerolineae;o__Ardenscatenales;f__Ardenscatenaceae |
|  | k__Bacteria;p__Chloroflexi;c__Anaerolineae;o__GCA004;f__ |
|  | k__Bacteria;p__Chloroflexi;c__Anaerolineae;o__H39;f__ |
|  | k__Bacteria;p__Chloroflexi;c__Chloroflexi;o__Chloroflexales;__ |
|  | k__Bacteria;p__GOUTA4;c__;o__;f__ |
|  | k__Bacteria;p__Planctomycetes;__;__;__ |
|  | k__Bacteria;p__Planctomycetes;c__;o__;f__ |
|  | k__Bacteria;p__Planctomycetes;c__OM190;o__CL500-15;f__ |
|  | k__Bacteria;p__Planctomycetes;c__Phycisphaerae;o__CCM11a;f__ |
|  | k__Bacteria;p__Planctomycetes;c__Pla3;o__;f__ |
|  | k__Bacteria;p__Proteobacteria;c__Alphaproteobacteria;o__Rhodospirillales;f__ |
|  | k__Bacteria;p__Proteobacteria;c__Deltaproteobacteria;o__NB1-j;f__NB1-i |
|  | k__Bacteria;p__Proteobacteria;c__Gammaproteobacteria;o__Alteromonadales;f__Alteromonadaceae |
|  | k__Bacteria;p__Tenericutes;c__Mollicutes;o__Acholeplasmatales;f__Acholeplasmataceae |
|  | k__Bacteria;p__Verrucomicrobia;c__[Pedosphaerae];o__[Pedosphaerales];f__OPB35 |
|  | k__Bacteria;p__WS2;c__SHA-109;o__;f__ |
|  | k__Bacteria;p__WS3;c__PRR-12;o__Sediment-1;f__CV106 |
|  | k__Bacteria;p__WS3;c__PRR-12;o__wb1_H11;f__ |
| **PNMP I** | k__Bacteria;p__Acidobacteria;c__Holophagae;o__Holophagales;f__Holophagaceae |
|  | k__Bacteria;p__Actinobacteria;c__Actinobacteria;o__Actinomycetales;f__Tsukamurellaceae |
|  | k__Bacteria;p__Chloroflexi;c__Ktedonobacteria;o__Thermogemmatisporales;f__ |
|  | k__Bacteria;p__Cyanobacteria;c__Oscillatoriophycideae;o__Oscillatoriales;f__Phormidiaceae |
|  | k__Bacteria;p__Firmicutes;c__Clostridia;o__Clostridiales;f__Peptococcaceae |
|  | k__Bacteria;p__Proteobacteria;c__Betaproteobacteria;o__Neisseriales;f__Neisseriaceae |
|  | k__Bacteria;p__Proteobacteria;c__Deltaproteobacteria;o__Desulfobacterales;f__Desulfobulbaceae |
|  | k__Bacteria;p__Spirochaetes;c__[Leptospirae];o__[Leptospirales];f__Sediment-4 |
|  | k__Bacteria;p__Spirochaetes;c__Spirochaetes;o__Spirochaetales;f__Spirochaetaceae |
|  | k__Bacteria;p__Synergistetes;c__Synergistia;o__Synergistales;f__Dethiosulfovibrionaceae |
|  | k__Bacteria;p__Verrucomicrobia;__;__;__ |
| **PNMP II** | k__Bacteria;p__Actinobacteria;c__Actinobacteria;o__Actinomycetales;f__Intrasporangiaceae |
|  | k__Bacteria;p__Bacteroidetes;c__Bacteroidia;o__Bacteroidales;f__Rikenellaceae |
|  | k__Bacteria;p__Bacteroidetes;c__Cytophagia;o__Cytophagales;f__[Amoebophilaceae] |
|  | k__Bacteria;p__Chlamydiae;c__Chlamydiia;o__Chlamydiales;f__Simkaniaceae |
|  | k__Bacteria;p__Chloroflexi;c__SHA-26;o__;f__ |
|  | k__Bacteria;p__Cyanobacteria;__;__;__ |
|  | k__Bacteria;p__Cyanobacteria;c__Nostocophycideae;o__Nostocales;f__Scytonemataceae |
|  | k__Bacteria;p__Firmicutes;c__Bacilli;o__Bacillales;f__Thermoactinomycetaceae |
|  | k__Bacteria;p__Firmicutes;c__Clostridia;o__Clostridiales;f__Lachnospiraceae |
|  | k__Bacteria;p__Firmicutes;c__Clostridia;o__Clostridiales;f__Veillonellaceae |
|  | k__Bacteria;p__Proteobacteria;c__Alphaproteobacteria;o__BD7-3;f__ |
|  | k__Bacteria;p__Proteobacteria;c__Alphaproteobacteria;o__Rhizobiales;f__Xanthobacteraceae |
|  | k__Bacteria;p__Proteobacteria;c__Alphaproteobacteria;o__Rickettsiales;__ |
|  | k__Bacteria;p__Proteobacteria;c__Deltaproteobacteria;o__Myxococcales;f__0319-6G20 |
|  | k__Bacteria;p__Proteobacteria;c__Gammaproteobacteria;o__Legionellales;f__Legionellaceae |
|  | k__Bacteria;p__Proteobacteria;c__TA18;o__CV90;f__ |
|  | k__Bacteria;p__Proteobacteria;c__TA18;o__PHOS-HD29;f__ |

Supplementary Table 9: Differential abundance analysis was performed using the Wilcoxon test. The values presented correspond to the p-values calculated for each sample and location.

<https://drive.google.com/drive/folders/16KxdFER6eCRq-5xcOJIcNrh5bjSrHLS2?usp=sharing>
